# Recovery from Cogwheel Rigidity and Akinesia and Improvement in Vibration Sense and Olfactory Perception following Removal of an Epoxy-Oleic Acid DNA Adduct

**DOI:** 10.1155/2017/3512353

**Published:** 2017-10-18

**Authors:** Jean A. Monro, John McLaren-Howard, Mussadiq Shah, Peter O. O. Julu, Basant K. Puri

**Affiliations:** ^1^Breakspear Medical Group, Hemel Hempstead, Hertfordshire, UK; ^2^Acumen, P.O. Box 129, Tiverton, Devon, UK; ^3^Barts and the London School of Medicine, Queen Mary University of London and the William Harvey Research Institute, London, UK; ^4^Department of Medicine, Imperial College London, London, UK

## Abstract

The epoxy fatty acid *cis*-12,13-epoxy-oleic acid, which acts as a DNA adduct, may be generated during long-term storage of many seed oils, including those used in cooking, with frying oils and fried foods being a major source in the modern human diet. Removal of this epoxy fatty acid from the locus of the *N*-formyl peptide receptors was associated with recovery from cogwheel rigidity and akinesia as well as with improvement in vibration sense and olfactory perception.

## 1. Introduction

A DNA adduct is the covalent binding of a substance to DNA. The effect would depend on the position of the adduct on the genome. At a gene location, expression of the gene may be suppressed. On the promoter region of a gene, overexpression is a possibility.

We present the first case to be published of how removal of an epoxy-fatty acid from the locus of the *N*-formyl peptide receptors was associated with recovery from cogwheel rigidity and akinesia as well as with improvement in vibration sense and olfactory perception.

## 2. Case Report

A 51-year-old man presented with a two- to three-month history of episodes of akinesia affecting his hands. It was noted that his employment entailed regular and frequent flights, both long haul and short haul. The history was otherwise unremarkable.

Physical examination revealed cogwheel rigidity in the upper limbs and impaired vibration sense which was absent in the lower limbs, reduced rostrally to the level of the anterior superior iliac spine. He was positive on both the Unterberger test and Romberg's test. Important negative neurological signs were no evidence of abnormality of gait, no tremor, no nystagmus, and no pain. Clinically, his long-term memory appeared to be excellent. The clinical examination was otherwise unremarkable.

Routine blood tests were insignificant. Investigation for DNA adducts by analysis of genomic DNA from leucocytes by gas-liquid chromatography showed the presence of epoxy-oleic acid, which was localised to the *N*-formyl peptide receptors (FPRs) using DNA microarrays. Other investigations were carried out to exclude other possible causes of the patient's symptomatology; these proved negative and included testing for gonorrhoea, hepatitis B core antibodies, hepatitis B surface antigen, hepatitis C antibodies, HTLV-1, IgG and IgM antibodies to syphilis, and antibodies to chlamydia species, as well as the HIV duo (HIV-1 and HIV-2 p24 antigen) and Venereal Disease Research Laboratory (VDRL) tests.

Treatment was given in the form of supportive and detoxification nutrients, including glutathione, alpha-lipoic acid, methylcobalamin and phosphatidylcholine, and regular high-temperature Iratherm® sessions. A repeat DNA adduct investigation a year later proved negative. Supportive nutritional treatment continued, and the patient was assessed, a year later, for olfactory and gustatory function and then again four months later. During this four-month period, his score on the University of Pennsylvania Smell Identification Test [1] rose from 37/40 (a normal healthy score is 35/40) to 38/40. Similarly, his score on the Sniffin' Sticks identification at higher concentration test showed an increase from 11/12 (normal) to the maximum score of 12/12 (above normal). Meanwhile, the patient became free of the cogwheel rigidity and akinesia, while sensory vibration impairment became limited to the left lower limb.

## 3. Discussion

Epoxy-fatty acids occur naturally in some plant seed oils, but their toxic nature generally excludes those oils from human food uses. The *cis*-12,13-epoxy-oleic acid, also known as vernolic acid, considered in this paper is present in the seed oils of *Vernonia anthelmintica* and *Vernonia galamensis*. In the latter case, it can account for as much as 60 percent of the total fatty acids. Small amounts of both hydroxyl and epoxy fatty acids can be generated during long-term storage of many seed oils, including oils used for culinary purposes; frying oils and fried foods are the major source of *cis*-12,13-epoxy-oleic acid in the human diet [2]. This includes heated olive and sunflower oils [3]. Epoxy-fatty acids have been shown to be absorbed by healthy women [4]. The means by which an epoxy-fatty acid can adduct to genomic DNA has been explored in detail [5–7]. It is well established that DNA adduct formation with some potentially dangerous chemical intermediates can be promoted by epoxy-group molecules [8].

At the time of writing, three FPRs are known; FPR1, FPR2, and FPR3 are located in close proximity at 19q13.3. While most members of the G-protein-coupled FPR family are now known to be expressed by murine vomeronasal sensory neurons [9], the above case report suggests that FPRs may also have a role in early Parkinson's disease. Indeed, the amyloid-beta peptide Aβ_42_ has been shown to be an agonist for FPR-like-1 (FPRL1); FPRL1 is strongly expressed by inflammatory cells infiltrating neuritic plaques in postmortem Alzheimer cerebral tissue and so may be involved in the cerebral inflammatory processes associated with this form of dementia [10].

As this report is based on a single case, there are clearly limitations. A much larger study needs to be carried out in order to ascertain the extent of the association of DNA adducts with the neurological symptomology described above. The conclusions which may be drawn regarding the efficacy of the described treatment for the DNA adducts are also limited by the fact that neither the patient nor his doctors were blinded to the nature of his treatment and the fact that there was no control group which received placebo treatment.

This case highlights the clinical value of checking for and removing DNA adducts and calls attention to the need for further research into the possible roles of FPRs in neurodegenerative diseases.

## 4. Conclusion

In conclusion, this case report demonstrates that the presence of DNA adducts should be considered in cases of neurological presentations involving movement and/or perceptual disorders when no other causes can be found. Furthermore, the presence of such DNA adducts need not be permanent; removal may be associated with clinical improvement.

## Abbreviations

Aβ_42_: Amyloid-β_42_
DNA: Deoxyribonucleic acid
FPR: *N*-formyl peptide receptor
HIV: Human immunodeficiency virus
HTLV-1: Human T-lymphotropic virus 1
IgG: Immunoglobulin G
IgM: Immunoglobulin M
VDRL: Venereal Disease Research Laboratory.
